# Editorial: Effects of dietary nutrients on intestinal microbiome: insights into gastrointestinal diseases in animals

**DOI:** 10.3389/fcimb.2024.1466495

**Published:** 2024-09-30

**Authors:** Jing Gao, Kang Xu, Jie Yin, Gabriele Brecchia

**Affiliations:** ^1^ Hunan Academy of Forestry, National Engineering Research Center for Oil Tea, Changsha, China; ^2^ Institute of Subtropical Agriculture, Chinese Academy of Sciences, Changsha, China; ^3^ College of Animal Science and Technology, Hunan Agricultural University, Changsha, China; ^4^ Department of Veterinary Medicine, University of Milan, Lodi, Italy

**Keywords:** dietary nutrients, intestinal microbiome, gastrointestinal diseases, plant extracts, probiotic, prebiotics

We conducted this Research Topic to inventory what we presently know about diet-gut microbial-animal health. One of the main factors influencing an alteration in bacterial flora is nutrition, and the various elements of diet have a significant impact on the gut microbiota (Lee et al., 2022). Consequently, it is critical to fully comprehend if and how certain dietary components affect host health. A significant number of the effects of nutrition on human health and disorders are mediated or altered by gut microbiota. The complex ecosystem of microorganisms known as the human gut microbiota occupies the gut tract and plays a crucial role in defending host metabolism and immunity (Kayama et al., 2020). The majority of the advantages provided by the intestinal microbiota to the physiology of the host pathophysiology are closely linked with the metabolism of gut microbes. In terms of relative genetic content, bacteria account for the greatest portion of these contributions to ecosystem functioning. The direct advantage that is generally provided by microbial metabolism to the host may be attributed to the exogenous and endogenous substrates, considering that the substrates of gut microbiota could fulfil the host nutritional requirements. Interestingly, chronic diet-related diseases have clearly demonstrated the critical roles that microbe-host- immunity interactions play in coordinating diet-mediated host health and diseases, with the majority of these diseases closely linked to both the gut microbiota and host immunity (Alvarez et al., 2021). Consequently, researching possible relationships between nutrition, gut microbiota, and animal health is crucial. The present Special Topic of Frontiers in Cellular and Infection Microbiology offers an important step to learning more about the subjects listed above. Our Research Topic includes eight primary research articles.

Nutrient absorption and host defense against external stimuli depend heavily on the gut. Due to their high prevalence and debilitating clinical symptoms, inflammation-related intestinal diseases like enteritis, inflammatory bowel disease, and colorectal cancer pose a significant burden to human health. Interestingly, the use of plant extracts in intestinal diseases is increasing and results have shown that many plant extracts show positive effects in inhibiting intestinal diseases. Liang et al. recently studied whether the raw Broussonetia papyrifera leaf (BP) without fermentation could alleviate intestinal inflammation in dextran sodium sulfate (Sigurdsson et al., 2023)-treated mice considering its good *in vitro* performance against intestinal pathogens including *Clostridium perfringens, Salmonella Typhimurium, and Salmonella enterica*. They found that, although the efficacy of BP leaf extract in reducing intestinal inflammation generated by DSS was not demonstrated as expected, *Faecalibaculum* and *Akkermansia* species of intestinal beneficial microbes were found to be more abundant following a single BPE administration. Moreover, macleaya cordata extract possesses anti-inflammatory, antioxidant, and antibacterial properties, making it a viable substitute for antibiotics. Wu et al. found that microbiome-transcriptome analysis indicated that nutritional supplementation with macleaya cordata extract significantly changed several immunological pathways but had no effect on microbiota structure. More importantly, Xiasangju has a reputation for cleaning the liver, improving vision, removing heat, and aiding in detoxifying, and Xiasangju has been proven to have antiviral, antibacterial, anti-cancer, antioxidant, immune-modulating, and liver-protective qualities in recent studies. Sun et al. indicated that the concentration of *Lactobacillus* johnsonii was dramatically enhanced in diets containing 1% Xiasangju residue, while the relative abundance of *Weissella jogaeotgali* was significantly increased in diets containing 2% and 4% Xiasangju residue. The relative abundances of *Treponema porcinum* and *Escherichia coli* were dramatically reduced by nutritional supplementation with 0.5%, 1.0%, 2%, and 4% Xiasangju residue. Likewise, another Chinese traditional medicine, Danggui Shaoyao San, could boost cognitive and learning capacities by lowering the quantity of negative gut bacteria (Jin et al.).

It has been demonstrated that taking probiotic supplements enhances immunity and overall health. And probiotics could improve the homeostasis of internal microbiota to enhance human intestinal health. For instance, Zhang et al. studied Bacillus coagulans by switching the reduction in amounts of *Enterococcus, Clostridium*, and *Lactobacillus* in the jejunum and increasing the amount of *Escherichia coli* caused by ETEC K88, as well as *Bifidobacterium, Lactobacillus*, and *Enterococcus* in the colon. The modification in food intake had a favorable influence on the gut microbiome imbalance. Wang et al. demonstrated that high-fat diets significantly increased the concentration of *Acetobacter malorum* in the intestines, leading to a notable inflammatory reaction through the bacteria's re-colonization. This finding indicates that remodeling the gut microbiota plays a major part in determining the form of an inflammatory response in distant tissues.

Prebiotics are also instruments for managing the microbiota that enhance host health by acting specifically on the gastrointestinal tract through the gut. Ge et al. proved that, when fed with 0.02% sodium butyrate, the species abundance of Chinese soft-shelled turtle significantly increased, especially the relative abundance of *Clostridium sensu stricto*. Zhou et al. suggested that alginate oligosaccharide can alter the composition of intestinal microbes by decreasing harmful microbes including *Streptococcus* and *Prevotellaceae*_NK3B31_group and increasing *Enterobacter*, a beneficial bacterium.

## Conclusions

Experiments on gut microbiota and their relation to intestinal diseases are continuing. Research on the connection between diets and gut microbiota is making progress in analyzing how nutrients affect host immunity and health. Our RT on the latest developments in the diet-gut axis is current because there is not yet a comprehensive report that compiles the advancements that have occurred in this field in recent years. The scientific community studying intestinal disorders may find the RT articles, which cover a variety of topics related to current nutrition research, to be a valuable resource. This could potentially be used as a roadmap for creating innovative approaches to animal health research.
